# TERT promoter mutations in melanoma survival

**DOI:** 10.18632/oncotarget.26688

**Published:** 2019-02-22

**Authors:** Eduardo Nagore, Sivaramakrishna Rachakonda, Rajiv Kumar

**Affiliations:** Department of Dermatology, Instituto Valenciano de Oncologia, Valencia, Spain; School of Medicine, Universidad Catolica da Valenciana “San Vincent Martir”, Valencia, Spain; Division of Molecular Genetic Epidemiology, German Cancer Research Center, Heidelberg, Germany; Consortium for Translational Research, German Cancer Research Center, Heidelberg, Germany

**Keywords:** TERT promoter, survival, mutations, melanoma, prognostic

In melanoma, the telomerase reverse transcriptase (TERT) promoter mutations, that have emerged as the prominent somatic alterations, associate with the markers of poor patient outcome [1]. In combination with BRAF/NRAS mutations, the noncoding TERT promoter mutations in primary tumors were shown to result in poor disease-free and melanoma-specific survival [2]. The extension of the study to measure the effect of individual TERT promoter mutations, unexpectedly, resulted in the discovery that the less frequent -138/-139CC > TT tandem mutation associated with the worst disease-free and melanoma-specific survival in stage I and stage II patients. Of the three main TERT promoter alterations, the presence of the -146C > T mutation in tumors had the least effect on patient survival. The effect of the -138/-139CC > TT tandem mutation was particularly enhanced in combination with the BRAF/NRAS mutations, which can have a potential clinical implication for patients treated with MAP kinase inhibitors [3].

**Figure 1:**
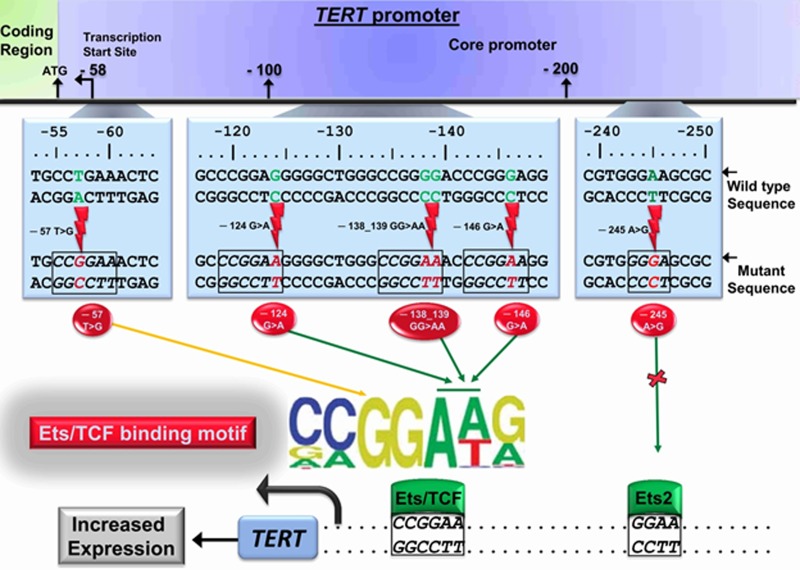
Schematic representation of the TERT promoter mutations The mutations at the -57, -124, -138/-139 and -146 bp position from ATG start site of the TERT gene create CCGGAA/T consensus sites for ETS transcription factors. The binding of the ETS transcription factors in conjunction with preexisting native ETS sites in the proximity causes epigenetic transformations and recruitment of pol II leading to mono-allelic TERT expression. The variant allele of the polymorphism represented by rs2853669 at the -245 bp position abrogates the preexisting non-canonical ETS2 site.

The promoter mutations were discovered through sequencing of a disease segregating locus in a multi-generational melanoma pedigree resulting in identification of the causal germline A > C (T > G) mutation at -57 bp from ATG start site of the TERT gene [4]. Subsequent screening of tumors from unrelated melanoma patients led to the discovery of the frequent somatic -124C > T and -146C > T TERT promoter mutations that create CCGGA/T binding sites for E-twenty-six (ETS) transcription factors similar to the germline mutation in the melanoma family (Figure 1) [4]. The -57A > C mutation has also been occasionally reported as a somatic alteration. Binding of ETS transcription factors to the *de novo* sites created by the promoter mutations leads to enhanced TERT transcription and consequent telomerase rejuvenation that imparts tumor cells with infinite proliferative capability [1]. Further studies showed that the somatic TERT promoter mutations, besides melanoma, were frequent in many cancers, particularly those that arise from tissues with low rate of self-renewal [5]. Presence of the TERT promoter mutations was also shown to effect disease-outcome and patient survival in many other cancers [1]. In melanoma and bladder cancer, the presence of the variant allele of the T > C polymorphism, that abrogates a preexisting non-canonical ETS site, at the -245 bp position represented by rs2853669 in the TERT promoter was reported to modulate the effect of the TERT promoter mutations (Figure 1) [1].

With the exception of skin cancers, the -124C > T mutation in different cancers is the most frequent TERT promoter alteration; however, in skin cancers, the -146 C > T is more frequent than the -124 C > T mutation. Skin cancers are further characterized by CC > TT tandem mutations at -124/-125 and -138/-139 bp position, which also create the CCGGAA/T consensus moieties (Figure 1) [1]. While negligible in non-skin cancers, about 10 percent of tumors in melanoma patients with the TERT promoter mutations carry the -138/-139CC > TT tandem base-change. The nucleotide change at -139 bp position is also a rare polymorphism represented by rs35550267; an acquired C > T base change at -138 bp position in conjunction with the variant allele at -139 bp position would result in the observed tandem mutation. However, enhanced frequency in skin cancers in the absence of the polymorphism points to the ultraviolet radiation etiology for the -138/-139CC > TT tandem mutation [3].

Theoretically, the binding of ETS transcription factors at the sites created by the TERT promoter mutations should have similar biological impacts. That concept is challenged by the observation of two-fold higher TERT expression in tumors with the -124 C > T mutation than in tumors with the -146 C > T alteration [1]. The multimeric ETS factor GABP binding at *de novo* mutant sites leads to switch from an inactive H3K27me3 to active histone H3K4me2/3 mark, recruitment of pol II and induction of mono-allelic TERT transcription [6]. Genetic disruption of a GABPβ1L, a tetramer-forming isomer of GABP isomer has been demonstrated to result in the promoter mutation dependent TERT silencing leading to telomere loss and cell death [7]. GABP complex binding to the *de novo* sites created by the TERT promoter also involves preexisting native ETS sites, hence a possibility of the intra-mutational differences. Binding of ETS1 specifically at the site created by the -146C > T TERT promoter mutation involving non-canonical NF-kB signaling has also been demonstrated [8]. Introduction of the TERT promoter mutations in pluripotent stem and neural precursor cells abrogates the usual transcriptional repression of TERT upon differentiation; however, at stem cell level the presence of only -124C > T mutation results in modest increase in TERT transcription without an upsurge in telomerase activity [9]. Despite similarity of the consensus moieties created by the different promoter mutations, experimental data, thus, reveal subtle differences, which extend to the effect on patient survival as observed in melanoma with -138/139CC > TT TERT promoter mutation in tumors [3].

Biological effect of the -138/-139CC > TT TERT promoter mutation, unlike the two common somatic alterations, has remained uninvestigated. An earlier study showed that the TERT promoter mutations promote tumorigenesis by immortalization and genomic stability in two phases [10]. The TERT promoter mutations extend cellular proliferation by stabilizing shortest telomeres and critically short telomeres in turn result in genomic instability. It is thus possible that the -138/-139CC > TT tandem mutation promotes greater genomic instability and has less effect on TERT upregulation than the other two promoter mutations, hence, poor patient survival. Further investigation of differences between different TERT promoter mutations, so far the most common noncoding genetic alterations in human cancers that affect tumorigenesis through altered expression, will help in refined understanding of the role of those alterations for possible clinical interventions.
